# Imaging features associated with H3 K27-altered and H3 G34-mutant gliomas: a narrative systematic review

**DOI:** 10.1186/s40644-022-00500-3

**Published:** 2022-11-17

**Authors:** Arian Lasocki, Gehad Abdalla, Geoffrey Chow, Stefanie C. Thust

**Affiliations:** 1 Department of Cancer Imaging, Peter MacCallum Cancer Centre, Grattan St, Melbourne, Victoria 3000 Australia; 2 Sir Peter MacCallum Department of Oncology, The University of Melbourne, Parkville, Victoria Australia; 3 Department of Radiology, The University of Melbourne, Parkville, Victoria Australia; 4 Lysholm Department of Neuroradiology, National Hospital for Neurology and Neurosurgery, London, UK; 5 Department of Radiology, Mansoura University Hospital, Mansoura, Egypt; 6 Department of Radiology, Royal Free Hospital, London, UK; 7 Department of Brain Repair and Rehabilitation, Neuroradiological Academic Unit, UCL Institute of Neurology, London, UK

**Keywords:** Magnetic resonance imaging, Radiogenomics, H3 K27M-altered glioma, H3 G34-mutant glioma

## Abstract

**Background:**

Advances in molecular diagnostics accomplished the discovery of two malignant glioma entities harboring alterations in the H3 histone: diffuse midline glioma, H3 K27-altered and diffuse hemispheric glioma, H3 G34-mutant. Radiogenomics research, which aims to correlate tumor imaging features with genotypes, has not comprehensively examined histone-altered gliomas (HAG). The aim of this research was to synthesize the current published data on imaging features associated with HAG.

**Methods:**

A systematic search was performed in March 2022 using PubMed and the Cochrane Library, identifying studies on the imaging features associated with H3 K27-altered and/or H3 G34-mutant gliomas.

**Results:**

Forty-seven studies fulfilled the inclusion criteria, the majority on H3 K27-altered gliomas. Just under half (21/47) were case reports or short series, the remainder being diagnostic accuracy studies. Despite heterogeneous methodology, some themes emerged. In particular, enhancement of H3 K27M-altered gliomas is variable and can be less than expected given their highly malignant behavior. Low apparent diffusion coefficient values have been suggested as a biomarker of H3 K27-alteration, but high values do not exclude this genotype. Promising correlations between high relative cerebral blood volume values and H3 K27-alteration require further validation. Limited data on H3 G34-mutant gliomas suggest some morphologic overlap with 1p/19q-codeleted oligodendrogliomas.

**Conclusions:**

The existing data are limited, especially for H3 G34-mutant gliomas and artificial intelligence techniques. Current evidence indicates that imaging-based predictions of HAG are insufficient to replace histological assessment. In particular, H3 K27-altered gliomas should be considered when occurring in typical midline locations irrespective of enhancement characteristics.

**Supplementary Information:**

The online version contains supplementary material available at 10.1186/s40644-022-00500-3.

## Introduction

Advances in molecular diagnostic methods have improved the distinction of brain tumors based on characteristic genetic abnormalities, which has been reflected in the 2016 update to the World Health Organization (WHO) Classification of Tumors of the Central Nervous System (forthwith referred to as WHO 2016) and the more recent 2021 WHO Classification (forthwith WHO 2021). WHO 2016 introduced the entity *diffuse midline glioma, H3 K27M-mutant*, which typically occurs in children and young adults, in characteristic midline locations (in particular, thalamus, brainstem and spinal cord) [1]. A midline location is critical for the diagnosis of this neoplasm, and hence the diagnosis cannot be applied to tumors which demonstrate an H3 K27M mutation but occur elsewhere in the brain [2]. Subsequently, gliomas with a similar demographic distribution characterized by an H3 G34 mutation have been identified, however these typically arise in the cerebral hemispheres, not in the midline [3, 4]. Growing understanding of these tumors has led to a diagnostic refinement in WHO 2021 [4], in which the two groups are now *diffuse midline glioma, H3 K27-altered* and *diffuse hemispheric glioma, H3 G34-mutant*, respectively. Both are classed WHO grade 4 *pediatric-type diffuse high-grade gliomas* [4].

In parallel with our growing understanding of the molecular mechanisms underlying gliomagenesis, research has correlated imaging features, in particular MRI, with key genetic alterations, known as “radiogenomics” or “imaging genomics”. Given their much higher incidence, the majority of this research has examined adult gliomas, predominantly targeting two key genetic markers, IDH mutations and 1p/19q-codeletion (combined loss of the short arm of chromosome 1 and the long arm of chromosome 19) [1, 5], which are absent in histone-altered gliomas (HAG). Earlier radiogenomics research has utilized conventional imaging assessment (“conventional radiogenomics”), while more recent work has investigated augmentation with artificial intelligence (AI) techniques (“AI radiogenomics”), including radiomics [6] and deep learning.

Radiogenomics arguably has greater potential value in HAG than in adult-type gliomas. This is particularly the case for the H3 K27-altered group, given that their midline location increases the morbidity risk associated with obtaining a definitive tissue diagnosis. Because of their rarity and recent discovery, large radiogenomics studies exploring features of HAG are currently limited, and much of the existing literature consists of case reports and short series. The lower incidence of HAG also makes research into AI-augmented diagnostic methods particularly challenging. The purpose of this systematic review was to summarize the existing imaging literature on HAG, with a view to identifying diagnostic trends and targets for future research.

## Materials and methods

This research was performed based on the Preferred Reporting Items for Systematic Reviews and Meta-Analyses (PRISMA-DTA) criteria for diagnostic accuracy studies [7]. Acknowledging the limited number of larger series published at this time, case reports and short case series were also examined, but exempt from PRISMA-DTA.

### Data sources

A systematic search was performed in March 2022 using PubMed and the Cochrane Library, identifying all relevant papers published at the time of the search. The following search key words were used: (brain tumor OR brain tumour OR glioma OR midline glioma OR diffuse midline glioma OR pontine glioma OR DIPG OR brain neoplasm OR brain cancer OR glioblastoma) AND (magnetic resonance imaging OR imaging) AND (histone OR histone-mutant OR mutant OR mutation OR gene OR H3 OR G34 OR K27M OR H3 OR H3.1 OR H3.3 OR K27M OR H3 G34). The search was deliberately broad, rather than explicitly searching for particular techniques, in order to avoid biasing some techniques over others.

### Study selection

The abstracts of all articles retrieved in the initial search were screened independently by two reviewers (board-certified radiologists with research experience in neuro-oncology). All selected full text manuscripts were reviewed independently by the same two reviewers. The exclusion criteria were: no imaging interpretation; animal or laboratory measurements only; study confined to technical comparison between different MRI acquisition technique(s); studies restricted to predicting WHO histological grade or light microscopic features by imaging; or no English full-text. The major inclusion criterion was: contains a description of imaging features associated with diagnosis and/or prognosis of one or more histone-altered glioma subtypes as defined in WHO 2016 or WHO 2021 (based on the search terms described above). References for all studies fulfilling the above criteria were checked, and if additional publications potentially met the criteria, these were also assessed against the exclusion and inclusion criteria as outlined above. Case reports with or without literature review were included, provided that imaging findings were described. In cases of disagreement, each full text article was reviewed by a third (senior) reviewer and the discrepancy was resolved by consensus.

### Data analysis

The results of the included studies were documented with the use of a data extraction form to derive the study methods, study population, glioma mutation(s) identified, imaging findings, correlations and statistical results. Greater detail regarding the data extraction table is presented in Table 1. Each of the reviewers independently performed the full-text screening followed by the data extraction with two reviewers analyzing each publication. Discrepancies were resolved in consensus with a third (senior) reviewer.

**Table 1:** Data extraction table

Author, year	Study design	Main research purpose	Study population (N, age, gender)	WHO grade(s)	Location	Glioma mutation	MRI sequence(s) analysed	Imaging features described with key statistical results (if any)
Lober RM, 2014 [8]	Retrospective	Using DWI to stratify DIPG subsets with distinct clinical behavior	• *n* = 1 histone mutant / 20 DIPG • 14 yo • M	G3, G4 (NOS for target patient)	Pons/periventricular nodule	H3-K27M	T1wC+, DWI	• Periventricular nodular enhancement • Baseline ADC high = 1393. Follow up ADC mixed = 1172 & 1875. Periventricular lesion: 728
Ishibashi K, 2016 [9]	Case report	To report a pediatric midline glioma with H3F3A K27M mutation before and after malignant transformation	• *n* = 1 • 14 yo • M	G2 on initial biopsy; G3 on resection	Thalamus	H3-K27M	FLAIR, T1wC+	• Non-enhancing • Tumor dissemination at 12 months after diagnosis
Aboian MS, 2017 [10]	Retrospective	Characterize imaging features of DMGs with H3 K27M mutation and determine any specific imaging features correlate with histone mutation	• *n* = 24 histone mutant /*n* = 33 DMGs • Mean age: 108.5 months • 17 M / 7F	Not stated	Pontine/cerebellar peduncle (11), vermis (4), subcallosal (1), thalamic (6), cervical spine (2)	H3-K27M	T2w, FLAIR, T1w, T1wC+, DWI	• 15/24: cystic components or necrosis, 4/24: oedema • 16/24 (67%) enhanced “heterogenous or uniform” • 5/24 multifocal, 18/24 infiltrative, 4/24 mass effect, 18/24 irregular border, 6/24 CSF based metastases, 9/24 direct cortical invasion • No difference between imaging characteristics of histone mutant and wild type gliomas
Yoshimoto K, 2017 [11]	Case series	Examine the prevalence and clinicopathological features of H3F3A and G34R/V mutant high grade gliomas	• *n* = 14 histone mutant (*n* = 10 K27M, *n* = 4 G34R) • Mean age G34R: 10.5 yo; K27M: 15 yo • G34R: 2 M:2F; K27M: 6 M:4F	Not stated	G34R: parietal, multiple lobes, thalamus	H3-K27M, G34R and G34V	T2w, FLAIR, T1w, T1wC+, DWI	• Hyperintense • Absent or faint then marked enhancement (2/4 cases) after tumour progression • G34R: case 1: gliomatosis cerebri growth pattern. Case 2: high density on CT + intratumoral calcification.Central restricted diffusion. Case 3: low density on CT + intratumoral calcification, indefinite tumour margins on MRI
Lopez GY, 2017 [12]	Case series	Describing 2 cases of DMG with mosaic expression of H3.3 K27M mutant protein and its implications with regards to classification and grading.	• *n* = 2 • 30 yo and 69 yo • 1 M and 1F	1) G4 2) G3	Bilateral thalamus	H3 K27M mutant, IDH wild type, ATRX (one lost, another retained)	T1wC+	• Heterogeneously enhancing (both)
Vettermann FJ, 2017 [13]	Retrospective	Whether malignant tumor progression as observed for IDH-mutated gliomas can also be found in K27M midline gliomas	• *n* = 14 • Median age:21 yo • 8 M, 6F	G2–4	Thalamus (5), brain stem (4), spinal cord (2), mesial temporal (2), cerebellum (1)	H3K27M, ATRX, IDH, FGFR1	T2w, T1w, T1wC+	• 2/14 showed contrast enhancement on follow up, during progression, after 13.1 (case 2) and 3.8 m (case 10). • FET-PET: Maximal tumor-to-background ratio (TBR) - 6/8 with follow up scans ‘significant’ increase in TBR (> 20%)
Gilbert AR, 2018 [14]	Case report	A case of pineal region DIPG in a child (2nd case of pineal region DIPG overall)	• *n* = 1 • 12 yo • F	G4	Pineal gland	H3-K27M	FLAIR, T1w, T1wC+, MRS	• Hyperintense • Heterogeneous and rim enhancing, with posterior fossa and spinal CSF dissemination on follow up • Hypodense on CT / hydrocephalus. • MRS: suppressed NAA peak and elevated choline, lipid, and lactate peaks
Vettermann FJ, 2018 [15]	Case series	Characterize the imaging features of H3-G34 mutant gliomas using MRI and 18F-FET PET.	• *n* = 8 • Median age: 27 yo • 2 M, 6F	G3 (*n* = 2), G4 (*n* = 5), 1: NOS	Multifocal (2), rest: lobar/ thalamus/ truncus corporis callosi	H3-G34R (all)	T2w and/or FLAIR, 3D T1w, 3D T1wC+	• 4/8 cystic components, 0/8 significant perifocal oedema • 5/8 heterogeneous enhancement • 1/8 hemorrhage and calcification. • 8/8 high uptake intensity on 18F-FET PET with a median TBRmax of 3.4 • Dynamic PET (7/8) short median TTP min = 12.5 minutes
D’Amico RS, 2018 [16]	Case report	Present clinical and pathological features of pineal region GBM	• *n* = 1 histone mutant / 8 GBM • 38 yo • M	G4	Pineal region	H3-K27M, IDH-wild, lost ATRX	FLAIR, DWI, T1w, T1wC+	• Enhancing • Diffuse seeding of the ventricular system with enhancing tumor • Hydrocephalus
Daoud EV, 2018 [17]	Case series	Characterize adult brainstem gliomas, focusing on the H3-K27M radiologic features and clinical outcome	• *n* = 7 histone mutant • Median age: 41 yo • 6 M and 1F	Low grade (*n* = 2), high grade (*n* = 5)	Midbrain tectum (1), midbrain tegmentum (1), pons (2), medulla (1), cerebellar peduncle/pons (2)	H3-K27M, ATRX-retained, p53	FLAIR, T1C+	• 1/7: T2/FLAIR hyperintense • 2/7 exophytic, 3/7 hydrocephalus • 6/7 enhancing: 2/7 heterogeneous, 1/7 minimal amorphous, 1/7 focal, 1/7 poor irregular, 1/7 “enhancing” • No correlation between contrast enhancement and H3-K27M status, p = 0.1
Dormegny L, 2018 [18]	Case report	Improve accuracy of spinal cord biopsies and analyzing clinical, radiological and surgical features	• *n* = 1 • 21 yo • M	G4	Dorsal thoracic cord T9–11	H3-K27M, IDH (wild-type)	T2w, T1w, T1wC+	• T2 hyperintense; 3 weeks later (no intervention) - heterogeneous • Loss of T2: intralesional hemorrhage • Initial - localized contrast uptake at T10; 3 weeks later (no intervention) - multiple areas of extended contrast uptake form T9 to conus (T12-L1), with nodular uptake in cauda equina
Gao Y, 2018 [19]	Case report	Report a case of DMG with histone H3-K27M mutation which simultaneously showed PNET-like appearance	• *n* = 1 • 51 yo • F	Not stated	Cervical cord C5–7	H3-K27M, IDH wild type, ATRX retained	T2w, T1w, T1wC+	• Hyperintense • Mild enhancement
Puntonet J, 2018 [20]	Retrospective	Correlate the histological and radiological features of G34R mutant high-grade gliomas	• *n* = 12 • Mean age: 16 yo (range = 6–31 yo), • 9 M and 3F	G4 (6), G3 (4), HGG NOS (2)	All supratentorial, predominant temporal lobe (7), basal ganglia (4), all leptomeningeal contact, meningeal invasion (1), ependymal contact (9)	All H3K27M, G34R, GFAP +ve and BRAFv600e wild-type. ATRX lost (9). P53 + ve (9).	T2, FLAIR, T1wC+ (11 patients), DWI (4), PWI (2), T2*w (6)	• T2 hyperintense (7), T2 isointense (5), T2* hypointense (4/6). • Enhancement: 4/11 intense, 5/11 minimal to moderate, 2/11 no enhancement. 3/12 nodular, 3/12 patchy, 3/12 serpiginous, 3/12 ring-like • 3/3 restricted diffusion (ADC ~ 4800.10 − 6 mm2/s) • 8/10 precontrast T1 hyperintensity, 4/12 no necrosis, 4/12 minimal necrosis, 4/12 high necrosis. • 6/11 no oedema, 4/11 minimal oedema, 1/11 moderate oedema. • 2/2 ASL-PWI hyper perfusion (relative CBF: 1.74–2.85). • 2/2 CT hyperdense
Jung JS, 2019 [21]	Retrospective	Evaluate the imaging characteristics of DMG with H3 K27M mutation in the spinal cord + evaluate predicting the presence of H3 K27M using a machine learning–based classification model.	• *n* = 24 histone mutant / *n* = 41 spinal cord gliomas • Mean age: 35.5 yo • 17 M / 7F	Not stated	Axial location (18/24 central, 6/24 eccentric); Longitudinal location (8/42 cervical, 14/24 thoracic, 2/24 lumbar)	H3-K27M	T2w, T1w, T1wC+, GRE T2*w	• 21/24 hyperintense, 3/24 isointense, 1/24 hypointense; 14/24 edema • 8/24 diffuse enhancement, 9/24 partial, 7/24 none; 5/17 irregular rim enhancement • 6/24 hemorrhage, 4/24 necrosis, 3/24 tumoral cyst, 1/24 syringohydromyelia • 16/24 ill-defined margin • 10/24 CSF-based tumor spread • Significant difference in tumoral hemorrhage between H3-K27M and wildtype, p = 0.003 • RF model: sensitivity = 45.8%, specificity = 88.2%, AUC = 0.63, 95% CI (0.45–0.80), accuracy = 63.4%
Qiu T, 2019 [22]	Retrospective	To summarize the imaging characteristics of adult H3 K27M-mutant gliomas	• *n* = 66 histone mutant • Age range: 20–60 yo • 40 M and 26F	Not stated	Thalamus (38), brainstem (6) (2 pons, 4 medulla, all dorsal); cerebellum (2); thalamus (2), whole brain (8), corpus callosum (3), hypothalamus (1), spinal cord (4) (1 in each of cervical, cervicothoracic, thoracic, and lumbar cord), cerebral hemispheres (2)	H3-K27M	T2w, FLAIR, T1w, T1wC+ (in 61/66), DWI	• 10/66 peritumoral oedema • 11/61 none enhancing, 25/61 partial enhancement, 25/61 diffuse (≥ 50% of the whole lesion) • 1/66 hemorrhage; all (4/4) spinal cord lesions spanned 3 or more segments • 8/66 leptomeningeal dissemination • 66/66 unremarkable or moderate restriction.
He P, 2019 [23]	Case report	A rare H3 K27M–mutant glioblastoma in the hypothalamus.	• *n* = 1 • 27 yo • F	Not stated	Hypothalamus, intrasellar, suprasellar	H3-K27M, ATRX &p53& olig2. IDH wild-type	T2w, T1w, T1wC+	• T2 hyperintense • CT: isodense solid mass, local recurrent mass, high rCBV (not mentioned values) • Strongly heterogeneous enhanced solid lesion and nonenhanced cystic lesion • Local recurrent mass: low ADC (1.2806)
Chanchotisatien A, 2019 [24]	Case report	Presenting a slow- growing thalamic glioma with H3-K27M mutation.	• *n* = 1 • 39 yo • M	G2	Thalamus	H3-K27M, ATRX, IDH-wild	T2w, FLAIR, T1wC+	• T2 hyperintense • No enhancement on followup
Chen H, 2019 [25]	Retrospective	Noninvasively identify MRI markers predictive of H3 K27 M mutational status in diffuse midline tumors.	• *n* = 19 / *n* = 38 DMGs • Mean age: 27 yo • 11 M and 8F	G2 (*n* = 4), G3 (*n* = 5), G4 (*n* = 10)	Thalamus (12), midbrain (1), pons (2), corpus callosum (2), cerebellum (1), hypothalamus (1),	H3-K27M	T2w, FLAIR, T1w, T1wC+, DWI (in 31/38)	• Shape: 12/19 round/oval, 7/19 irregular • 4/19 solid, 15/19 cystic; 3/19 hemorrhage, 7/19 edema, multifocal 9/19 • Tumour margin: 8/19 sharp, 11/19 blurred • 6/19 intra-tumoral rim enhancement, 1/19 marginal, 4/19 heterogeneous, 8/19 none or minimal • DWI in H3-K27M: • Minimal ADC = 0.734 ± 0.120, Ratio of minimal ADC = 0.972 ± 0.165, • Peritumoral ADC = 0.937 ± 0.156, Ratio of peritumoral ADC = 1.240 ± 0.232. • Significant difference in Histone mutant vs wild type in: Younger age (*p* = 0.009), Minimal ADC (*p* = 0.020), Peritumoral ADC (*p* = 0.018), Ratio of minimal ADC (*p* = 0.018), Ratio of peritumoral ADC (*p* = 0.013) • Thresholds: Minimal ADC 0.728, Ratio of minimal ADC 0.982; Peritumoral ADC 1.004, Ratio of peritumoral ADC 1.248 • Combination of DWI parameters: sensitivity = 88.89%, specificity = 76.92%, AUC = 0.872 (0.75–1.00)
Aboian MS, 2019 [26]	Retrospective	Identify differences in imaging diffusion characteristics between H3- K27M mutant and wild-type DMGs	• *n* = 23 • Mean age: 8.9 yo • 14 M and 9F	Not stated	Pons/vermis/4th ventricle (17), thalamus (5), subcallosal (1)	H3-K27M	DWI and ADC map values, FLAIR used for ROI registration	• No significant differences in ADC mean, median, minimum, and percentile values between histone mutant and wild-type gliomas
Miyazaki T, 2019 [27]	Case report	Present first case of DMG with H3-K27M mutation in a pregnant woman followed by fatal hemorrhage during the postpartum period.	• *n* = 1 • 26 yo • F (pregnant)	Not stated	Thalamus/midbrain	H3K27M, IDH-wild	T2w, FLAIR T1w, T2*w, T1wC+, DWI, MRA	• T2 hyperintense, poorly circumscribed • not enhanced • CT: isodense, hydrocephalus
Karlowee V, 2019 [28]	Retrospective	Analyze the EZH2 expression level and revealed its association with the poor survival of patients with H3K27M mutant-positive tumors.	• *n* = 12 • Age range: 6–56 yo • 8 M and 4F	Not stated	Distant recurrence (9), thalamus (9), pons (1)	H3-K27M, ATRX (4), p53 (8), EZH2 (9)	T2w, FLAIR, T1w, T1wC+, DWI	• 12/12 hyperintense, 2/12 cyst formation • Enhancement: 5/12 partly, 2/12 homogeneously, 3/12 heterogeneously, 2/12 none • 4/12 intratumoral hemorrhage, 9/12 dissemination + distant recurrence • 10/12 high DWI, 1/12 iso, 1/12 low DWI • No relationship between immunohistochemical staining results and clinical or imaging characteristics.
Giagnacovo M, 2020 [29]	Retrospective	Assess consistency between DIPG histo-molecular findings and clinical-radiological features.	• *n* = 19 / *n* = 22 DIPGs • Mean age: 8 yo • 10 M and 9F	G4 (*n* = 9), G3 (*n* = 8), G2 (*n* = 2)	Pons (at least 50% and causing expansion)	H3F3A/HIST1H3B K27M	T2w, FLAIR, T1w, T1wC+ +/− DWI, PWI, DTI, MRS	• Unsharp margins and patchy enhancement in one image for histone mutant
Chiang J, 2020 [30]	Retrospective	Identify variables that correlated with the clinical diagnosis of aDIPG (atypical) and evaluate the consistency of radiographic diagnosis of aDIPG	• *n* = 12 histone mutant / *n* = 33 aDIPGs, • Median age: 9 yo • 6 M and 6F	G1–4	Extra pontine extension (3), eccentric within the pons (7)	H3F3A/HIST1H3B K27M, TP53	T2w, T1w, T1wC+, DWI +/− FLAIR, T2wC+, SWI/T2*w	• 3/12 ring enhancement • K27M: 10/12 ill-defined tumor margin, 7/12 eccentricity within pons, 3/12 extra pontine extension • No significant difference in imaging (or clinical) features of K27M vs wild-type
Garibotto F, 2020 [31]	Retrospective	Compare the clinical behavior of DMGs H3 K27M- mutant and non-histone mutant midline HGGs in NF1 vs. non-syndromic children and to report imaging features of NF1 HGGs.	• *n* = 1 H3-K27M/ *n* = 2 NF1 DMGs; *n* = 11 H3-K27M / *n* = 16 Non-NF1 DMGs • Age: NF1 - 11yo; Non-NF1 - range 3-16yo • NF1: 1 F; Non-NF1: 4 M, 7F	G4 (all)	NF1: pons/midbrain (1); Non-NF1: thalamus (4), pons (4), medulla (1), diencephalon-mesencephalon junction (2)	H3.3 (H3F3A) K27M	T2w, FLAIR, T1w, T1wC+, DWI, FLAIRC+, (MRS in 2 described cases)	• One described case (NF1 H3K27M lesion): • Irregular rim enhancement, adjacent expansile region without enhancement • Irregular central necrotic area in ponto-mesencephalic portion, adjacent expansile portion no necrosis • reduced diffusivity in ponto-mesencephalic portion (minimum absolute ADC value: 0.69 × 10^−3^ mm^2^/s) • Leptomeningeal dissemination • Single voxel MRS: Cho/NAA peak-height ratio: 3.44 and Cho/Cr ratio: 2.91
Tu JH, 2020 [32]	Case report	Report a case of H3 K27M mutant diffuse midline glioma with cartilaginous metaplasia	• *n* = 1 • 56 yo • F	Not stated	Medulla	H3-K27M, GFAP, IDH-wild, lost ATRX, negative BRAFV600E	T2w, T1w, T1wC+	• T2 heterogeneous, cystic degeneration • Ring enhancement • CT: irregular calcification
Fujioka Y, 2020 [33]	Case report	Report a case of DMG, H3 K27M mutant that mimicked a hemispheric malignant glioma in an elderly patient.	• *n* = 1 • 66 yo • F	Not stated	Bilateral thalamus, left hippocampus, and fronto-parietal lobes	H3-K27M, IDH wild-type, negative BRAFV600E, retained ATRX, GFAP	FLAIR, T1wC+	• Hyperintense • Multiple isolated enhancing lesions. Enhancement disseminated to lateral ventricles
Cheng Y, 2020 [34]	Case series	Examine the prevalence and clinic pathological features of H3F3A and G34R/V mutant HGGs	• *n* = 3 • 15 yo, 15 yo, 28 yo • 2 M and 1F	Not stated	Cases 1 and 2:frontal, case 3: temporal	1) & 3) H3.3 G34V, 2) H3.3 G34R,	T2w	• Variable, homogenous / heterogenous high • Enhancement not stated • Local recurrence in all cases (after 10 or 5 months of 1st operation)
Baroni LV, 2020 [35]	Case series	Report three brainstem tumors with an initial indolent course that later developed classical imaging and clinical features of DIPG.	• *n* = 3 • 3 yo, 11 months, 6 yo • 2 M and 1F	1) Not stated, 2) G4, 3) G4	1) ponto-medullary junction, 2) medulla/pons, 3) brainstem and the pontocerebellar angle	all H3.3 K27M, 2 cases with TP 53	FLAIR, T2w	• All cases: hyperintense • 1) Non-enhancing, 2) non-enhancing, 3) not stated • All cases show increase in size in follow up (range 11 months to 5 years)
Babarczy K, 2020 [36]	Case report	Report one of the oldest patients having so far been reported with an immunohistochemically confirmed DMG, H3 K27M-mutant	• *n* = 1 • 73 yo • F	G4	Cervical cord (C2) extending to medulla, pons, cerebral peduncles, internal capsules bilaterally and right pallidum	H3-K27M, GFAP positive, IDH-wild	T2w, FLAIR, T1w, T1wC+, DWI, MRA, STIR (spine), MRS	• FLAIR hyperintense • No enhancement • No diffusion restriction • MRA: normal • MRS: high metabolism (no values)
Lu VM, 2020 [37]	Case report	Illustrate H3 K27M mutation occurring in cortically-based diffuse gliomas not midline structures and discuss the uncertainties regarding grading and prognostic classification for such tumours.	• *n* = 1 • 9 yo • F	Not stated	Pons	H3-K27M	T1wC+	• Not stated but enhancement highlighted in image • Progression in 2 months time (intracranial/intraspinal metastases)
Su X, 2020 [38]	Retrospective	Investigate the feasibility of predicting H3 K27M mutation status using an automated machine learning	• *n* = 40 histone mutant / *n* = 100 midline gliomas • Mean age: 23.6 yo • 14 M and 26F	G4	Midline (not otherwise specified)	H3-K27M	T2w, FLAIR, T1w, T1wC+	• 10 important features, including 3 shape, 3 first-order, 2 GLSZM, 1 GLCM, and 1 GLDM, were included. • Tumor shape features important in predicting H3 K27M mutation, maximum 2D diameter of the slice of H3 K27M–mutant tumors was smaller than that of wild-type (41.31 vs 59.35, *P* = 0.007) • Sensitivity range of 10 models: 0.55–0.73 • Specificity range of 10 models: 0.57–0.93 • AUC range for 10 models (0.73–0.90)
Chiba K, 2020 [39]	Retrospective	Investigate the correlation between the original site of thalamic gliomas and patients’ clinical outcomes retrospectively and to determine appropriate treatment strategies.	• *n* = 4 Histone mutant / *n* = 10 pediatric thalamic gliomas • Age range: 8–17 yo • 2 M, 2F	G3–4	All thalamopulvinar (TP)	H3-K27M	T2w, T1w, T1wC+, DWI (2/4), methionine-PET (2/4), DTI (1/4)	• T2 hyperintense • 1/4 heterogeneous enhancement, 1/4 mixed faint and homogeneous enhancement, 2/4 faint enhancement • 2/4 high DWI • The presence of H3 K27M mutation and TP location were closely related to each other (*p* = 0.0036)
Rodriguez Gutierrez D, 2020 [40]	Retrospective	To correlate imaging characteristics and outcome measures of pediatric patients with newly diagnosed non-brainstem HGG with pathologic and molecular data	• *n* = 23 H3K27M, *n* = 7 H3G34R / 113 gliomas • H3K27M: mean 12.1 yo; H3G34R: mean 13.1 yo; H3K27M: 11 M and 12F; H3G34R: 3 M and 4F	G3 (*n* = 21), G4 (*n* = 92)	Cerebral hemispheres and midline	H3F3a K27M, G34R	T2w, FLAIR, T1w, T1wC+	• Perilesional oedema: H3 K27M (10/23 none, 13/23 minor), H3 G34 (1/7 none, 3/7 minor, 2/7 moderate, 1/7 severe) • Necrosis: H3 K27M (16/23 yes, 6/23 no), H3G34 (4/7 yes, 2/7 no) • Enhancement: H3 K27M (14/23 strong, 1/23 moderate, 7/23 minor/none), H3 G34 (1/7 strong, 1/7 moderate, 4/7 minor/none) • Hemorrhage: H3 K27M (7/23 yes, 15/23 no), H3 G34 (4/7 yes, 2/7 no) • Tumor definition: H3 K27M (19/23 well-defined, 5/23 ill-defined/diffuse), H3 G34R (2 well-defined, 5 ill-defined/diffuse) • Versus Midline wildtype, H3 K27M–mutant tumors showed more enhancement (*P* < 0.05) and older age • ADC values were not significantly different between H3 K27M–mutant and Mid- line WT tumors or between H3G34R and cerebral wildtype. • Versus cerebral wildtype, tumor definition for H3 G34R mutants was significantly different, *p* < 0.05
Kay MD, 2020 [41]	Case report	Report rare extra cranial metastases from glioblastoma with PNET-like components and demonstrate the utility of FDG PET/CT for revealing distant metastases from glioblastoma	• *n* = 1 • 17 yo • F	G4	Temporal lobe	H3G34, IDH-wild	T2w, T1wC+, PET post resection	• T2 hyperintense • Heterogeneous enhancement • Presented with hematoma • Post resection: invasion into the left greater wing of the sphenoid, leptomeningeal drop and osseous metastases
Onishi S, 2020 [42]	Case series	Describing radiological and immunostaining characteristics of H3.3 G34R-Mutant Glioma	• *n* = 3 • Ages: 13, 19 and 15 yo 1 M and 2F	2/3 glioblastoma, 1/3 HGG	1) frontal, 2) parietal, 3) parieto-occipital	All: H3.3 G34R mutation, IDH-wild BRAF-wildtype, retained 1p/19q	T2w, FLAIR, T1w, T1wC+, DWI, MRS, ASL-PWI	• 3/3 T2/FLAIR moderately hyperintense • 1/3 poor enhancement 2/3 partial enhancement • 3/3 DWI hyperintense (ADC range = 0.625–0.810) • 3/3 mild edema. • 2/3 low tumor blood flow on ASL • 3/3: high choline peak, marked decrease in NAA peak, small lactate peak. • 3/3 iso to hyperdense mass without calcification
Thust S, 2021 [43]	Retrospective	To assess anatomical and quantitative diffusion-weighted MR imaging features in H3 K27M histone-mutant diffuse midline glioma	• *n* = 15 • Median age: 19 yo • 6 M and 9 F	G4	Midline brain	H3-K27M	T2w, FLAIR, T1w, T1wC+, DWI, DSC perfusion MRI (2/15)	• T2/FLAIR signal: 13/15 heterogeneous, 2/15 homogeneous. 2/15 with cysts • Margin: 8/15 distinct, 7/15 indistinct • Enhancement: 4/15 solid enhancement, 6/15 rim enhancing + necrosis, 3/15 none • DWI: • ADCmin in solid tumor = 0.84 (±0.15 SD) ADCmin/NAWM ratio = 1.097 (±0.149) • ADCmean in solid tumor = 1.12 (±0.25) ADCmean/NAWM ratio = 1.466 (±0.299) • Hemorrhage: 3/15 macro, 2/15 petechial • Hydrocephalus: 6/15 requiring shunting • 2/15 elevated rCBV ((5.9, 3.5) • 18F-choline PET (1/15): tracer accumulation • A significant difference (P = 0.01) between the 2nd centile of the volumetric ADC histogram and the ROI ADCmin values. • No statistical significance between ROI ADCmin and the 5th and 10th histogram percentiles, or ROI ADCmin/NAWM ratio and the ADC5th percentile/NAWM ratio or ROI ADCmean and the histogram ADCmedian and ADCmean or ROI ADCmean/NAWM ratio and the histogram ADCmean/NAWM ratio
Picart T, 2021 [44]	Retrospective	To describe the characteristics of DHG H3G34-mutant in adults and to compare them to those of established types of adult WHO grade IV gliomas	• *n* = 17 H3G34R • Mean age: 25 yo • 11 M and 6F	Not stated	H3.3G34R: frontal (11), parietal (11), temporal (3), occipital (1), corpus callosum (3), midline (4); H3.3K27M: 33/33 midline, 2 with temporal extension	17/17 H3F3a G34R, 14/16 TP53 positive, 13/14 loss of ATRX	T2w, FLAIR, T1w, T1wC+, MRS (9/17), DCE-PWI (8/17)	• H3.3 G34R: 9/16 poorly delineated/infiltrative, 3/16 Initial hemorrhage, 2/16 cyst, 1/16 necrosis; H3.3 K27M: 3/28 cyst, 10/28 necrosis • H3.3 G34R: 4/15 enhancement, 6/15 faint enhancement, 5/15 none; H3.3 K27M: 8/29 enhancement • H3.3 G34R: 12/13 restriction (8/13 focal); H3.3 K27M: 3/23 restriction • 4/8 elevated rCBV (> 1.7). 4/9 elevated Choline/N-Acetyl-Aspartate ratio > 2 and/or of lipid/ lactate peaks • H3 G34-mutant occurred in younger patients (*p* = .05), more frequently involved the parietal lobe (*p* < .001), more frequently presented as a hemorrhagic tumor at diagnosis (p = .03), less frequently demonstrated contrast enhancement (*p* = .05) and necrosis (*p* = .03), more frequently displayed ADC restriction (*p* < .001)
Cheng R, 2021 [45]	Case report	Report a pediatric patient with spinal cord H3 K27M-mutant DMG	• *n* = 1 • 7 yo • F	G4	Cervical cord C2–7 intramedullary	H3-K27M	T2w, T1w, T1wC+	• T2 slightly hyperintense • Heterogeneous enhancement
Li Q, 2021 [46]	Retrospective	MRI characteristics of brain DMG-histone mutant using radiomics	• *n* = 16 histone mutant / *n* = 30 DMGs, • Median age: 25.5 yo • 10 M and 6F	Not stated	10/16 thalamus, 6/16 brainstem	H3-K27M	T2w, T1w, T1wC+	• No detailed information. From single selected figure –T2 hyper intense • Faint enhancement • Cyst formation showed sig diff between H3K27M and WT (OR = 7.800, 95% CI 1.476–41.214; *p* = 0.024) • No statistical significance in: necrosis (*p* = 0.191), hemorrhage (*p* = 0.657) and T1/T2 ratio (*p* = 0.689)
Kandemirli S, 2021 [47]	Retrospective	Machine learning to predict histone mutation	• *n* = 50 histone mutant / *n* = 59 histone wild type • Median age: 10 yo mutant / 30.5 yo histone-wild	Not stated	Thalamus only (17), 12 center in thalamus (12), center in the pons (13), remaining posterior fossa structures (8)	H3K27M	T2w, T1w, T1wC+, FLAIR and ADC	• Median age in the H3K27M mutant group was significantly lower compared with the wild-type cohort • H3K27M-mutant and wild-type tumors show no significant difference in tumor size, enhancement, internal necrotic changes, or infiltrative appearance
Kathrani N, 2022 [48]	Retrospective	Assess DWI and DSC-PWI to predict the H3K27M mutation status in DMGs non-invasively	• *n* = 48 • Mean age: 23 yo • 21 M and 27F	G2–4	Thalamus (28), midbrain (6), pons (10), medulla (2), others (2)	H3-K27M	DWI, DSC (in 34/48)	• Peritumoral ADC = 1.1, nPT ADC = 1.64, min ADC = 0.76, nADC = 1.11 • rCBV = 25.17, nrCBV = 3.44, rCBF = 266.15, uncorrected nrCBV = 3.5 • Significantly lower PT ADC and nPT ADC and higher rCBV, nrCBV, rCBF, uncorrected nrCBV in histone mutant group • Thalamic subgroup analysis: H3K27M showed significantly lower min ADC PT ADC, nADC, nPT ADC and significantly higher nrCBV, rCBF, nrCBF, uncorrected rCBV, uncorrected nrCBV, and corrected rCBV **Predicting histone mutation:** • PT ADC (cut-off = 1.245; AUC 0.6, Sn 47%, Sp 79%), nPT ADC (cut-off = 1.853; AUC = 0.6, Sn 52%, Sp 77%), nrCBV (cut-off = 1.83; AUC = 0.6, Sn 46%, Sp 76%) and uncorrected nrCBV (cut-off = 2.28; AUC = 0.7, Sn 61%, Sp 71%), In thalamic subgroup: nADC (cut-off = 1.129; AUC = 0.7, Sn 61%, Sp 75%), PT ADC (cut-off = 1.185; AUC = 0.65, Sn 68, 68%), nPT ADC (cut-off = 1.853; AUC = 0.65, Sn 64%, Sp 71%), nrCBV (cut-off = 1.83; AUC = 0.703, Sn 47%, Sp 85%), nrCBF (cut- off = 2.73; AUC = 0.74, Sn 71%, Sp 75%), and uncorrected nrCBV (cut- off = 2.27; AUC = 0.759, Sn 60%, Sp 86%)
Ikeda K, 2022 [49]	Retrospective	Establish high intensity on DWI in non-enhancing tumors (DWI-Gadolinium mismatch sign) as imaging biomarker for H3K27M DMG	• *n* = 6 • Median age: 23 yo (range 6–31) • 4 M and 2F	Not stated	6/6 thalamus	H3-K27M, IDH-wild type	T2w, T2*, FLAIR, T1w, T1wC+, DWI	• T2/FLAIR hyperintense • 6/6 enhancement • 6/6 DWI high • 5/6 DWI-Gd mismatch sign positive • DWI-Gd mismatch sign and intratumoral bleeding present in thalamic gliomas than in pons with statistical significance (*p* = 0.046 and *p* = 0.0017)
Su X, 2022 [50]	Retrospective	Investigate the capacity of quantitative MRI in identifying the H3 K27M mutation status of DMG	• *n* = 23 • Age range = 6–47 yo • 13 M and 10F	G4	Juvenile group: hemispheric near midline (1), diencephalon (2), brainstem (8) adult group: diencephalon (3), brainstem (9)	H3K27M	T2, FLAIR, DWI, T1w, T1wC+, PWI, MRS, DTI	• Juvenile group: rADC_M = 1.56, rADC_15th = 1.15, rADC_25th = 1.31, rADC_50th = 1.50, rADC_75th = 1.68, rADC_max = 2.94, Ins/tCr = 1.11, NAA/tCr = 0.62, Cho/NAA = 0.96, Cho/rCr = 0.65, tNAA/tCr = 0.63, Glx/rCr = 0.89. • Adult group: rADC_M = 1.5, rADC_15th = 1.07, rADC_25th = 1.15, rADC_50th = 1.33, rADC_75th = 1.60, rADC_max = 3.05, Ins/tCr = 0.80, NAA/tCr = 0.60, Cho/NAA = 1.11, Cho/rCr = 0.57, tNAA/tCr = 0.86, Glx/rCr = 0.97 • rADC_15th, rADC_25th, rADC_50th, and rADC_75th values were significantly lower in mutation group (*p* < 0.05) • Ins/tCr values were lower in the juvenile (p = 0.003) and the adult (*p* = 0.025) mutation subgroups • rMD_mean and rMD_25th/50th/75th values of the mutation group were significantly lower in the adult subgroup (*p* < 0.001) • rCBV_mean/25th/50th/75th were slightly higher and the rTTP_mean/50th were slightly lower in the adult mutant subgroup; no significant differences in other PWI metrics • No significant difference in tumor size (*p* > 0.05).
Hohm A, 2021 [51]	Retrospective	Describe and compare MRI of pediatric DMG with known H3 K27 mutation status including H3.1 and H3.3 K27M subgroups	• *n* = 52 • Age: 10.2y (range 1.25–17.75yo) • 26 M and 26F	Not stated	19/52 thalamus/basal ganglia, 0 midbrain tectum, 27/52 pons, 5/52 spinal cord, 1 other (medulla)	H3.1 K27M & H3.3 K27M	T2w, T1w, T1wC+	• T2 hyperintense (44), homogeneous (3), predominantly homogeneous (17), predominantly inhomogeneous (23), inhomogeneous (4). • Margins: well defined (3), moderately defined (23), ill-defined (21). • Necrosis: yes (27); Edema: yes (8); CSF dissemination: (2) • Enhancement: strong (19), intermediate (9), mild (7), no (12), predominantly inhomogeneous (18), inhomogeneous (10); ring enhancement (25) • DWI: DWI hyperintense (22), restriction (5), not restricted (12) • SWI: signal loss (7), hemorrhage: yes (18) • H3.1 younger than H3.3 (*p* = 0.004) • H3K27M more commonly arising in pons and thalamus/BG vs WT widely distributed (*p* = 0.001) • H3 K27M more often T2 hyper intense (44/47, p = 0.02), T1 inhomogeneous (19/46, *p* = 0.02) • Spinal tumors: There were no imaging characteristics that significantly differentiated the two molecular groups
Kim H, 2021 [52]	Retrospective/ one histone mutant case	Report 13 MMRD-associated (9 sporadic and 4 Lynch syndrome) primary brain tumors to determine clinic pathological and molecular characteristics	• *n* = 1 • 11 yo • M	G4	Thalamus (right)	H3-K27M, lost ATRX, IDH-wild type	T2w, T1w, FLAIR, T1wC+ (from images)	• Multiple enhancing solid and cystic masses
Kurokawa R, 2022 [53]	Case series	Review the demographic, clinical, and neuroradiological features of DHGs-G34m in 3 original cases	• *n* = 3 • Ages: 16, 22 and 19 yo • F/M/F	2/3 G4	1/3 frontal, 2/3 parietal	3/3 H3 G34R, 2/3 p53, 3/3 ATRX, 3/3 IDH wild type, BRAF -ve GFAP +ve	CT, T2w, FLAIR T1w, T2*W, T1wC+, DWI, SWI, PWI	• 3/3 T2 hyperintense, 2/3 leptomeningeal contact laterally • 2/3 patchy enhancement, 1/3 heterogeneous enhancement • 3/3 restricted diffusion, ADC values range = 0.53–0.8 • 2/3 hyperdense on unenhanced CT without calcification; 3/3 intratumoral hemorrhage on T2*; 1/3 elevated CBF in enhancing portion
Cheng L, 2021 [54]	Retrospective	Describe the clinical and radiological characteristics of primary spinal H3 K27M-mutant DMG and compare with the H3 K27 wild-type	• *n* = 28 • Age: 28.7 yo • 19 M and 9F	G1 (0), G2 (9), G3 (10), G4 (9)	Cervical (7), cervicothoracic (4), thoracic (11), thoracolumbar (5), holocord (1). Median involved segments = 3	H3K27M p53 (20) ATRX loss (8), Ki-67 >/= 20% (18), IDH-wild type (28)	T2w, T1wC+	• Enhancement: partial (12), diffuse (13); pial enhancement (24) • Ill-defined margin (25) • Edema (16), hemorrhage (4), cysts (4), necrosis (10), syrinx (6) • Fewer H3K27M have syrinx vs WT (*p* = 0.017) • No other imaging features showed statistical significance

*Abbreviation key: DWI* Diffusion weighted imaging, *DIPG* diffuse intrinsic pontine glioma, *T1wC+* T1 weighted imaging with contrast, *ADC* Apparent diffusion coefficient, *FLAIR* Fluid attenuation inversion recovery, *DMG* Diffuse midline glioma, *G2/3/4* WHO grade 2/3/4, *IDH* Isocitrate dehydrogenase, *T2W* T2 weighted imaging, *FET-PET* Positron emission tomography (PET) using O-(2-[18F] fluoroethyl-)-L-tyrosine, *MRS* Magnetic resonance spectroscopy, *GBM* glioblastoma multiform, *PNET* primitive neuroectodermal tumor, *DL-GNT* Diffuse leptomeningeal glioneuronal tumor, *NOS* not otherwise specified, *PWI* perfusion weighted imaging, *GRE* gradient echo, *rCBV* relative cerebral blood volume, *ROI* region of interest, *MRA* magnetic resonance angiogram, *DTI* diffusion tensor imaging, *SWI* susceptibility weighted imaging, *NF-1* Neurofibromatosis type-1, *STIR* Short Tau Inversion Recovery, *AUC* area under the curve, *HGG* high grade glioma, *FDG* fluorodeoxyglucose, *ASL* arterial spin labelling, *NAWM* normal appearing white matter, *DHG* diffuse hemispheric glioma, *DCE* dynamic contrast-enhanced, *MMRD* Mismatch repair-deficient

### Study quality assessment

The study quality was examined using the Quality Assessment of Diagnostic Accuracy Studies (QUADAS-2) instrument [55]. We evaluated concerns regarding applicability in three domains (patient selection, index test and reference standard) and the risk of bias in four different domains (patient selection, index test, reference test and timing). Each study was independently assessed for quality and potential bias by two reviewers. Disagreements were resolved by consensus with a senior reviewer. QUADAS-2 assessment was conducted on all original research, but is not applicable to case reports.

### Statistical analysis

Descriptive data are presented in form of a narrative synthesis, because of the heterogeneity of reported imaging features, assessment methods and lack of consistent quantification.

## Data synthesis

**Fig. 1:**
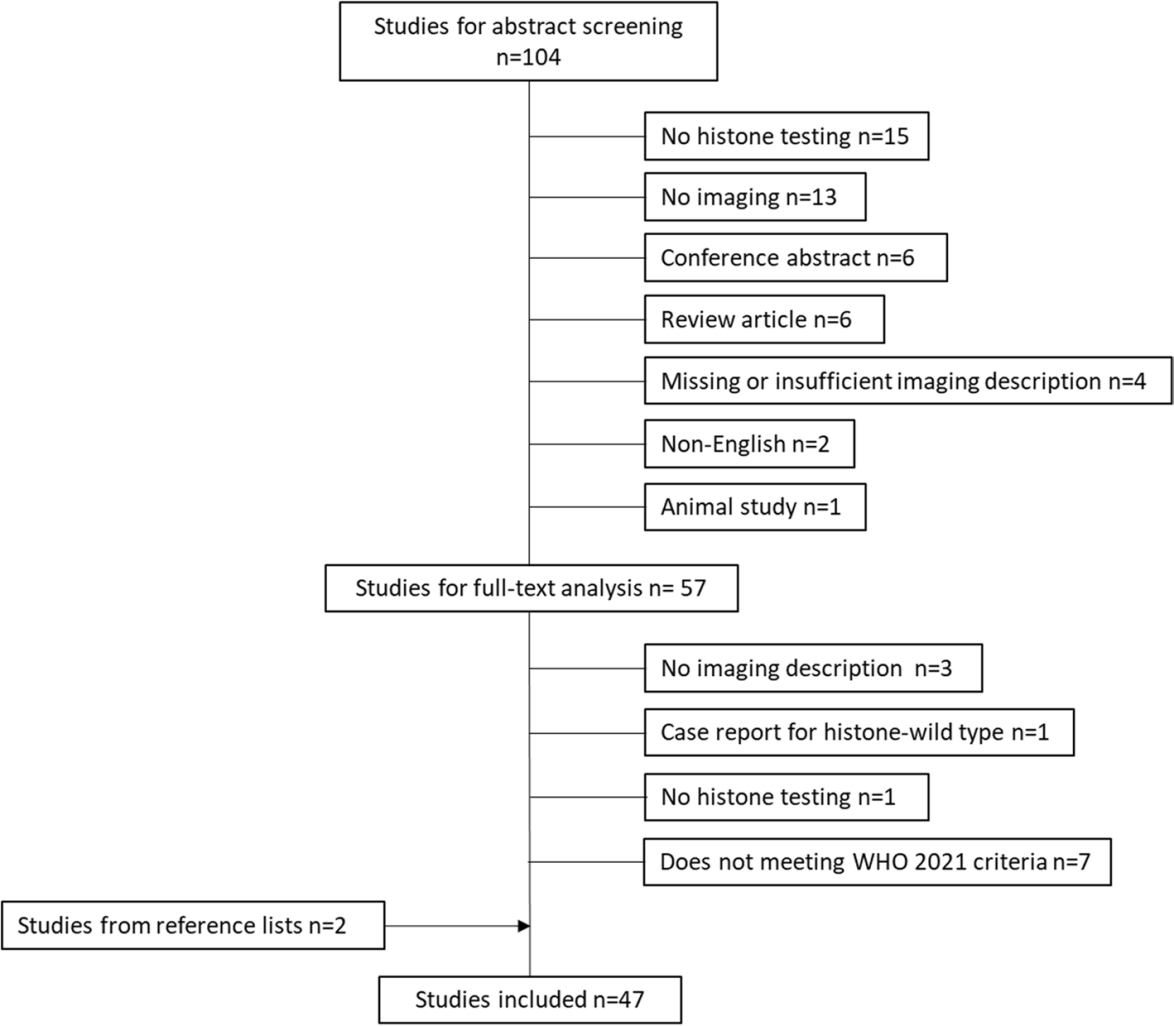
CONSORT diagram outlining included and excluded studies

A total of 47 papers was identified after exclusions [8–54] (Fig. 1). Just under half (21/47) of the included papers were case reports or short series (up to three cases). The majority of the publications (39/47) described only H3 K27-altered gliomas (typically reported as H3 K27M-mutant, reflecting the recency of the change in nomenclature), two described both H3 K27-altered and H3 G34-mutant gliomas, and six reports included only H3 G34-mutant gliomas (in one of these studies, K27-altered were included as a comparator, but were not the focus of the research [44]). The case reports generally described ‘novel’ features, for example previously undescribed tumor locations, clinical behavior, patient demographics or pathological features. Tumors varied between publications in terms of their histological grade. Despite the mostly high-grade nature of HAG, several tumors with grade 2 histology were described [9, 12, 24], highlighting that the lack of high grade histological features does not negate the need for appropriate molecular testing if the tumor occurs in a typical location [17] or demographic.

Studies assessing larger numbers of patients varied greatly in their method design. Patient demographics were heterogeneous, with studies variably assessing pediatric and/or adult patients. Several cohorts included only HAG, thus were unable to compare imaging appearances with H3-wildtype tumors in a similar location. The majority of studies assessed MRI appearances, with or without CT. Two series (one publication each for H3 K27-altered and H3 G34-mutant gliomas) assessed PET (positron emission tomography) using the amino acid tracer FET (fluorine-18-fluoroethyl-L-tyrosine) [13, 15]. Three recent papers assessed the use of MRI radiomics for predicting H3 K27 status [38, 46, 47].

## H3 K27-altered gliomas

### Patient demographics

As would be expected according to incidence, the likelihood of H3 K27 mutations varied depending on the age distributions within the study samples, with H3 K27-alteration being more common in younger age groups. For example, 19 of 22 DIPGs in the pediatric cohort reported by Giagnacovo et al. were H3 K27-altered [29], while only seven (28%) of 25 adult brainstem gliomas reported by Daoud et al. demonstrated H3 K27M mutations [17]. This is also well demonstrated by the cohort of diffuse midline gliomas reported by Chen et al., with H3 K27M-mutant patients being on average 15 years younger than -wildtype patients [25]; a similar finding was reported by Su et al. [50].

### Tumor location

Marked heterogeneity in reporting and study design limits assessment of the relative frequency of the different locations. Several studies assessed only tumors in specific locations, for example the pons (diffuse intrinsic pontine glioma, DIPG) or spinal cord, while others included only intracranial tumors. The reporting of tumor location was also variable, for example whether a pontine location was distinguished from other brainstem sites. Nevertheless, the thalami and brainstem (in particular pons) are unambiguously the most common locations. The spinal cord is the third-most-common location, though the data are limited.

There were notable differences in tumor location depending on the age group studied. The thalami were the most common location in cohorts which either largely or exclusively assessed adult patients [22, 25, 28, 46, 48]. In contrast, the brainstem was the most common overall location in studies targeting a pediatric population [10, 13, 31, 51]. Another common theme was that most intracranial H3 K27-altered gliomas were located in either the thalami or brainstem, while H3 K27-wildtype tumors were relatively more evenly distributed across midline locations [48, 51]. There are suggestions that the likelihood of H3 K27-alteration is higher in the brainstem rather than the thalami [48, 51], though this may relate to a brainstem location being more common in younger patients, who inherently have a higher likelihood of H3 K27-alteration. Two studies on spinal cord gliomas suggest that H3 K27M mutations occur in approximately half of cases, with 28/59 in one cohort [54] and 24/41 in another [21].

Beyond demonstration of a midline location, as a key diagnostic criterion [4], few anatomical characteristics have been described to predict H3 K27 status. One study by Chiba et al. subdivided their 10 pediatric thalamic gliomas (four H3 K27M-mutant) into three anatomical groups: anterior, combined thalamic and internal capsular, and thalamopulvinar [39]. All four H3 K27M-mutant gliomas in their cohort were thalamopulvinar, compared to only one H3 K27M-wildtype, and this association was statistically significant (*p* = 0.0036) despite the low number of cases [39]. Chiang et al. found slightly different rates of H3 K27M mutations in pontine tumors stratified as “typical” DIPG (defined radiographically as a poorly-demarcated, T1-hypointense and T2-hyperintense tumor with mass effect occupying ≥75% of the axial diameter of the pons; 50% H3 K27M-mutant), versus “atypical” DIPG (35%) and non-DIPG with an extrapontine epicenter (25%) [30]. Qiu et al. noted that all six of their H3 K27M-mutant gliomas which only involved the brainstem were located dorsally, though such tumors accounted for a minority of their cohort (6/66) [22]. In a cohort of spinal cord gliomas, neither the axial location (central vs eccentric) nor longitudinal location (cervical, thoracic or lumbar) correlated with H3 K27M status [21].

### Tumor margins and extent

Individual studies vary in their results, but both well- and ill-defined tumor margins may occur. Fewer studies have specifically assessed tumor size, though most H3 K27-altered appear to be relatively well demarcated. This is supported by the radiomics study of Su et al., which found that the maximal 2D slice diameter was significantly lower for H3 K27M-mutant gliomas compared to -wildtype tumors [38]. Nevertheless, these tumors can occasionally be larger. For example, out of 66 H3 K27M-mutant adult gliomas reported by Qiu et al., eight demonstrated cerebral hemispheric infiltration together with thalamic and/or brainstem involvement [22], and cases of extensive H3 K27M-mutant gliomas in older patients have been reported [33, 36].

Distant tumor spread was identified in several studies. For example, Karlowee et al. observed dissemination and remote recurrence in 75% of 12 H3 K27-altered gliomas [28]. Of the 66 H3 K27M-mutant gliomas described by Qiu et al., leptomeningeal and subependymal dissemination were noted in eight and three patients, respectively [22]. According to publications, such dissemination generally occurred later in the disease course rather than already being manifest at initial diagnosis, although details remained unclear. A midline location itself was associated with leptomeningeal dissemination [40], however, thus it is unclear whether the biology of H3 K27M-altered gliomas predisposes to leptomeningeal dissemination or whether this is simply related to their location. One case report described extracranial HAG metastases [41].

### Signal characteristics and contrast-enhancement

The data on signal characteristics, in particular contrast-enhancement, are highly variable, but it is clear that H3 K27M-altered gliomas demonstrate a spectrum of appearances, from a lack of enhancement to ring-enhancement with central necrosis [10]. Thus, a lack of enhancement should not dissuade from considering an H3 K27M-altered glioma. Hohm et al. found that H3 K27M-mutant gliomas in their pediatric cohort were more commonly T2-hyperintense and heterogeneous than H3 K27M-wildtype tumors [51]. A different pediatric study demonstrated significantly more enhancement in H3 K27M-mutant tumors than -wildtype (*p* < 0.05) [40]. However, other studies found no statistically significant differences in the degree of enhancement between H3 K27M-mutant and -wildtype tumors [10, 17, 54]. Information on the specific contrast agent, contrast dose and type of post-contrast T1-weighted imaging sequence(s) used is generally lacking.

### Hemorrhage

Results on the incidence of hemorrhage in H3 K27-altered gliomas are variable, but overall this feature seems to have limited predictive value. Hemorrhage was the only imaging feature predictive of H3 K27M mutation in a cohort of spinal cord gliomas, occurring in six of 24 (25%) H3 K27M-mutant gliomas, compared to none of the 17 H3 K27M-wildtype tumors (*p* = 0.033) [21]. However, in another cohort of 59 spinal cord gliomas, the rate of hemorrhage was almost identical (and marginally higher in the wildtype group); the presence of a tumor syrinx (being more common in H3 K27M-wildtype tumors) was the only MRI feature with a statistically significant difference in this study [54]. Similar variability has been reported intracranially, though no other studies have found a statistically significant difference in the rate of hemorrhage between H3 K27-altered and -wildtype tumors. Comparing across studies, there are suggestions that hemorrhage may be more common in pediatric patients than adults [22, 51], but this question has not been specifically investigated.

### Apparent diffusion coefficient values

Studies investigating apparent diffusion coefficient (ADC) values have reported variable results, similar to the variability in the conventional imaging appearances, with a recurring trend towards H3 K27-altered gliomas demonstrating lower ADC values. Chen et al. reported that both tumoral and peritumoral apparent diffusion coefficient (ADC) values were significantly lower in H3 K27M-mutant gliomas than -wildtype (ratio of minimal ADC and ratio of peritumoral ADC combined, AUC 0.872) [25]. Another study also found lower ADC values in the peritumoral region of H3 K27M-mutant gliomas [48]; ADC values within the tumoral region were lower in H3 K27M-mutant tumors located in the thalami, but this was not reproduced across their overall cohort [48]. A further study also noted that relative ADC histogram parameters (15th, 25th, 50th and 75th percentiles) were lower in the H3 K27M-mutant group [50]. In contrast, no statistically significant correlations between ADC values and H3 K27 status were identified in two other studies, one having calculated mean, median, minimum and maximum ADC values and percentiles [26], the other having examined average and minimum ADC values [40]. All of the 66 H3 K27M-mutant gliomas reported by Qiu et al. had low or moderate diffusivity, with none demonstrating diffusion restriction on visual inspection [22]. Thust et al. reported moderately low ADC values in some H3 K27M-mutant gliomas, consistent with previous findings in glioblastoma, but highlighted ADC variability [43].

### Other advanced MRI techniques

Two out of the 15 H3 K27M-mutant gliomas reported by Thust et al. were imaged with dynamic susceptibility contrast perfusion, both demonstrating elevated relative cerebral blood volume (rCBV; 3.5–5.9) [43]. Kathrani et al. reported higher rCBV in H3 K27M-mutant gliomas compared to -wildtype [48]. Su et al. noted slightly higher rCBV in their discovery cohort, but this was not replicated in the validation cohort [50]. The authors also evaluated several MR Spectroscopy parameters, with lower myo-inositol/total creatine values in the H3 K27M-mutant group being the only parameter with statistical significance [50]. A multivariate model developed from this research achieved AUC = 0.976 in the validation set, but this comprised only 13 patients [50], thus the reproducibility of this model is unknown.

### FET-PET

One study assessed the use of FET-PET in H3 K27-altered gliomas [13]. Baseline TBR_max_ (maximal tumor-to-background ratio) did not correlate with histological grade or patient outcome, but was potentially useful to identify a subsequent increase of > 20% in TBR_max_ which predicted tumor progression and poor survival [13]. However, in the case example provided, new contrast-enhancement coincided with the increase in TBR_max_ [13], hence the added diagnostic value of FET-PET is uncertain.

### Radiomics

Kandemirli et al. investigated radiomics for the prediction of H3 K27 status in a cohort of 109 tumors, comprising 50 H3 K27M-mutant and 59 -wildtype, with just over half being pediatric cases [47]. Of the two models investigated, better results were obtained using XGBoost with additional feature selection, which achieved an area under the curve (AUC) of 0.791 in the training set and 0.737 for the test set [47]. Su et al. examined a similar cohort, including 40 H3 K27M-mutant and 60 -wildtype midline gliomas across pediatric and adult age groups, using the Tree-based Pipeline Optimization Tool [38]. This study reported better results, with the best-performing of the 10 models assessed yielding AUC 0.903 in the training cohort and 0.85 in the validation set [38]. Of note, the latter results were obtained utilizing only the FLAIR sequence [38], while Kandemirli et al. incorporated multiple conventional sequences and ADC [47]. Li et al. used principal component analysis in a smaller cohort, comprising 30 tumors, of which 16 were H3 K27M-mutant [46]. They observed overlap between H3 K27M-mutant and -wildtype types, with only cyst formation (favoring a H3 K27M-mutant tumor) showing a statistically significant difference between the two [46]. All three of the above studies extracted features using PyRadiomics [38, 46, 47].

## H3 G34-mutant gliomas

Only eight studies reported on H3 G34-mutant gliomas, with small numbers. All cases were high-grade histologically, the majority grade 4 [11, 40, 42, 44, 53]. Yoshimito identified four G34-mutant tumors amongst 411 consecutive gliomas (1.0%) of all ages, compared to 10 H3 K27-altered gliomas [11]. Picart et al. also had fewer H3 G34-mutant gliomas than H3 K27-altered tumors in their cohort (17 compared to 32) [44]. In a pediatric cohort of gliomas divided into midline and cerebral hemispheric locations, H3 G34 mutations were demonstrated in seven of 54 cerebral cases [40].

### Tumor margins and location

All four of the H3 G34-mutant tumors reported by Yoshimoto et al. all had ill-defined tumor margins [11]. The gliomas varied in location, and some involved deeper structures such as the basal ganglia [11]. Five of the seven H3 G34-mutant tumors in a pediatric cohort were ill-defined, and tumor definition was significantly different to non-midline H3 G34-wildtype tumors (the majority being well-defined) [40]. Similarly, most of the 17 H3 G34-mutant gliomas reported by Picart et al. were ill-defined [44]. Midline involvement was observed in four of the patients in this cohort, but always as an extension of a primarily hemispheric tumor [44]. In contrast, two of the three H3 G34-mutant gliomas described by Kurokawa et al. were well-defined [53]. Similarly, in a series of 12 H3 G34-mutant gliomas, the tumors were most commonly large and well-delineated, with mild peritumoral edema [20]. Leptomeningeal contact was observed in all 12 [20]. Concordant with these results, the two H3 G34-mutant described by Onishi et al. exhibited little peritumoral edema given their large size [42].

### Contrast-enhancement

Eleven of the 17 H3 G34-mutant gliomas reported by Picart et al. demonstrated absent or faint contrast-enhancement initially, but all eight of these which received subsequent MRIs developed nodular or ring-enhancement after a median of 2.6 months [44]. Some other series have demonstrated relatively mild enhancement [11, 42], while a range of enhancement patterns have been reported in other cohorts [15, 20, 53]. As for H3 K27-altered tumors, there is limited information on the specific contrast agent, contrast dose and type of post-contrast T1-weighted imaging sequence(s) used.

### Other MRI features

Two of the four H3 G34-mutant tumors reported by Yoshimoto et al. demonstrated calcification [11]. One tumor in a cohort of eight reported by Vetterman et al. demonstrated both calcification and hemorrhage, while four demonstrated cystic components [15]. Microcalcifications have also been noted on histology [20]. All three tumors reported by Kurokawa et al. demonstrated intratumoral hemorrhage, with varying degrees of diffusion restriction [53]. Two tumors in one series had available arterial spin labelling perfusion data and both demonstrated hyperperfusion [20]. One small series described choline elevation and N-acetyl aspartate depletion on Spectroscopy [42].

### FET-PET

One study described FET-PET features of eight H3 G34-mutant gliomas, noting high uptake in all (median TBR_max_ 3.4, range 2.5–11.7) [15]. In contrast, the MRI appearances of these tumors were more variable; for example, three tumors did not demonstrate contrast-enhancement, while three demonstrated rim-enhancement with central necrosis [15].

## Data quality

**Fig. 2:**
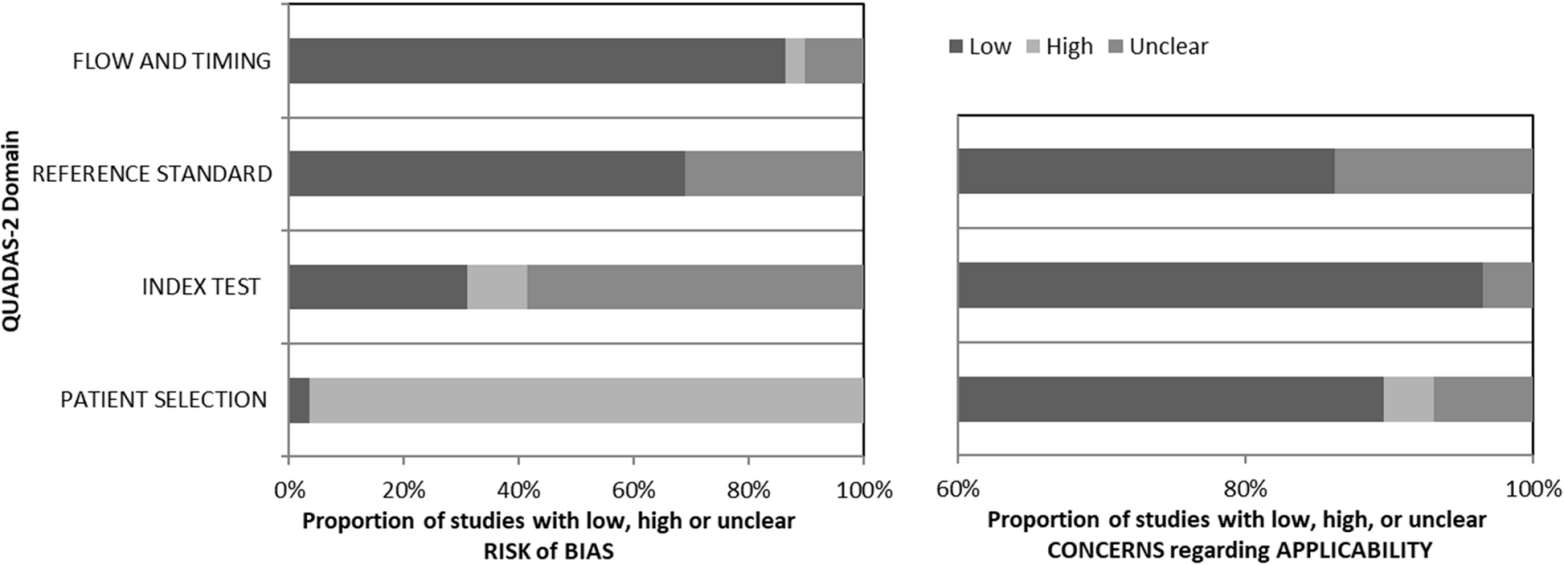
QUADAS-2 summary results

Of 47 included publications, 29 were diagnostic accuracy studies proceedable to QUADAS-2 assessment, with the remaining 18 studies being case reports or short series unsuitable for QUADAS-2 assessment. All studies were retrospective, introducing a high risk of bias in the patient selection domain, which parallels other radiogenomics literature. For most (*n* = 17) research, it was unclear whether the imaging was analyzed without knowledge of tissue results, specifically glioma genotypes, thus increasing the risk of bias. For 13 of the 29 publications, images were reviewed by only one observer or no information was provided at all. No formal interobserver comparisons were reported. The diagnostic reference standard was similar and judged to be appropriate in most (*n* = 20) studies. HAG genotype was presumed to represent a static tumor property, therefore the timing between reference standard and target test was considered appropriate for all studies. QUADAS-2 graphs are shown in Fig. 2, while individual study data are presented in Supplementary Material 1.

## Discussion

The reported cohort sizes are substantially lower for HAG than in the adult-type diffuse glioma radiogenomics literature, which is expected given their lower incidence, particularly for H3 G34-mutant gliomas. We identified marked heterogeneity of study designs, firstly in the cohorts investigated, but also for visual features assessed and in the definitions of such features, which limits comparability and precluded a meaningful meta-analysis of the data. Results have been conflicting for several features, highlighting that these tumors present a variety of appearances, whereby HAG cannot yet be confirmed or excluded with a high degree of confidence. The heterogeneity of the data indicates a need for more consistent biomarker definitions across studies, and highlights a challenge that could potentially benefit from AI approaches in future research. Despite these diagnostic limitations, some patterns have emerged, in particular for H3 K27-altered gliomas, which are summarized in Table 2. Of particular note, common to both H3 K27-altered and H3 G34-mutant gliomas was the frequent observation of less aggressive MRI appearances, belying their highly malignant histopathological classification.

**Table 2:** Features of H3 K27M-altered gliomas

Common locations	Less common locations	Tumor margins & extent	Enhancement	ADC values	rCBV
• Thalami (esp. adults) • Brainstem (esp. children) • Spinal cord	• Corpus callosum • Hypothalamus • Pineal gland • Tectum	• Usually well-defined and localized • Occasionally more widely infiltrative • Leptomeningeal dissemination common, esp. later in disease course	• Variable, but often less than expected for a high-grade tumor	• Variable, but generally low	• Variable, but generally increased

H3 K27-altered gliomas vary considerably in their degree of enhancement, and often demonstrate less contrast uptake than one would expect for a WHO grade 4 tumor. In contrast, the majority of adult-type grade 4 diffuse gliomas manifest as enhancing, centrally necrotic lesions [56]. Furthermore, a relative paucity of enhancement does not help distinguish between an H3 K27-altered glioma and a low grade adult-type diffuse glioma, which arguably is the more important distinction. Similar variability is evident in terms of tumor margins and ADC values. There have been some promising results with other advanced MRI features, in particular rCBV values, but data are currently limited and further research is warranted. Most H3 K27M-altered gliomas are relatively localized, though more diffusely infiltrative tumors (with a component of midline involvement) can occasionally be seen. Thus, the identification of thalamic and/or brainstem involvement in disseminated tumors could prompt testing for H3 K27-alteration, though the incidence would be expected to be low. There are possible differences in the imaging appearances of H3 K27-altered gliomas depending on their location. Suggestions that a pulvinar location in thalamic gliomas [39] or a dorsal location in pure brainstem gliomas [22] could predict H3 K27M mutation are notable, but require further validation. There is currently minimal information regarding whether a feature combination could provide additional predictive value. While the variability of MRI appearances limits the ability to confidently predict HAG genotypes, it highlights the importance of stereotactic biopsy and molecular testing for candidate lesions (e.g. based on location) even if the MRI appearances suggest a lower-grade tumor, for example based on well-defined margins or a lack of enhancement.

Subtle differences in the results between pediatric and adult studies have been reported. Most convincingly, a thalamic location is most common in adult patients [22, 25, 28, 46, 48], while a brainstem location is relatively more common in children [10, 13, 31, 51]. Beyond location, however, the data are less compelling, and there is clearly substantial overlap in the appearances. A particular challenge relates to methodological differences in the definitions of the assessed features, which make it difficult to compare across studies. In addition, studies combining pediatric and adult patients have generally not compared the two patient populations, and the limited patient numbers within each cohort present a further challenge. More targeted studies, correcting for patient age, would be required to clarify such observations.

The pre-test probability of an H3 K27-altered glioma varies according to each particular location, being highest in the brainstem, thalami and spinal cord. Data on less common midline locations are limited, but these seem to have a lower likelihood of H3 K27M-alteration. In turn, this will alter the role of features predictive of H3 K27M status, analogous to the difference in the ability to confidently predict an IDH mutation in adult-type diffuse gliomas depending on tumor grade (grade 2–3 vs grade 4) [5]. Thus, in a midline location with a higher likelihood of an H3 K27-altered glioma, a particular feature may allow more confident prediction of this genotype. In contrast, it may be difficult to confidently identify an H3 K27-altered glioma in a location with a lower pre-test probability, but instead the absence of features associated with H3 K27M-alteration could make it highly unlikely, such that definitive genetic testing would become redundant. This is particularly valuable given the challenging surgical access to many of these locations.

For G34-mutant gliomas, the existing data are scarce. A particular challenge is that the vast majority of hemispheric gliomas in adults will be H3 G34-wildtype. Nevertheless, some features worthy of further investigation have been reported. Tumors were often noted to be quite large, with relatively mild peritumoral edema. As for H3 K27-altered gliomas, H3 G34-mutant gliomas often demonstrate relatively mild enhancement given their WHO grade 4 status. For some tumors, there was possible morphologic overlap with IDH-mutant, 1p/19q-codeleted oligodendrogliomas: calcifications are characteristic of IDH-mutant, 1p/19q-codeleted oligodendrogliomas [5, 57, 58], but were reported in several H3 G34-mutant gliomas [11, 15]. Therefore, testing for an H3 G34 mutation should be considered for a calcified tumor without 1p/19q-codeletion in a young adult patient.

Very limited AI research exists on HAG. The results presented by Su et al. show promise, though the variability across the described models used raises the possibility over-fitting [38]. The substantial overlap in the features found in H3 K27M-mutant and -wildtype gliomas reported by Li et al. [46] is consistent with the results of conventional MRI radiogenomics studies, though the finding that cyst formation could predict H3 K27-alteration [46] is notable and warrants further investigation. A limitation of all three AI studies identified (and also some of the conventional MRI research) is that both pediatric and adult patients were included, in order to maximize numbers. This raises questions regarding clinical applicability, given that the H3 K27M-wildtype group will have included a mix of neoplasms. We expect that AI research in HAG will increase, but this may need to harness multi-institutional datasets in order to provide more uniform methodology whilst being relevant to clinical practice, for example when distinguishing between pediatric and adult patients and aiming to better characterize the tumors within the H3 K27M-wildtype group.

## Conclusion

The existing imaging data on HAG are limited and heterogeneous, but certain patterns have emerged. H3 K27-altered gliomas exhibit variable appearances, thus these tumors should be considered when occurring in typical locations irrespective of their conventional MRI appearances. Low ADC has been proposed as a biomarker of H3 K27-alteration, but results have been variable and facilitated diffusion does not exclude this malignant tumor type. Higher rCBV has also been reported in H3 K27-altered gliomas, but requires further validation. H3 G34-mutant gliomas are commonly large, with relatively mild peritumoral edema and variable, often mild enhancement. Some of these tumors may exhibit calcification, potentially mimicking IDH-mutant, 1p/19q-codeleted oligodendrogliomas. As a rare disease, HAG research will benefit from collaborative multi-institutional datasets, especially if investigating AI techniques. AI techniques could also be valuable for addressing the issue of heterogeneity of the existing data.

## Supplementary Information

**Additional file 1: Supplementary Material 1.** QUADAS-2 data for individual studies

## Data Availability

Not applicable.

## Abbreviations

WHO: World Health Organization
IDH: Isocitrate dehydrogenase
HAG: Histone-altered gliomas
AI: Artificial intelligence
PRISMA-DTA: Preferred Reporting Items for Systematic Reviews and Meta-Analyses
QUADAS-2: Quality Assessment of Diagnostic Accuracy Studies
PET: Positron emission tomography
FET: Fluorine-18-fluoroethyl-L-tyrosine
DIPG: Diffuse intrinsic pontine glioma
ADC: Apparent diffusion coefficient
rCBV: relative cerebral blood volume
TBR_max_: maximal tumor-to-background ratio
